# No Evidence for an Effect of the Smell of Hexanal on Trust in Human–Robot Interaction

**DOI:** 10.1007/s12369-022-00918-6

**Published:** 2022-09-15

**Authors:** Ilja Croijmans, Laura van Erp, Annelie Bakker, Lara Cramer, Sophie Heezen, Dana Van Mourik, Sterre Weaver, Ruud Hortensius

**Affiliations:** Centre for Language Studies, Radboud University, Erasmusplein 1, 6525 HT Nijmegen, The Netherlands

**Keywords:** Smell, Trust, Human–robot interaction, Collaboration, Hexanal, Replication

## Abstract

The level of interpersonal trust among people is partially determined through the sense of smell. Hexanal, a molecule which smell resembles freshly cut grass, can increase trust in people. Here, we ask the question if smell can be leveraged to facilitate human–robot interaction and test whether hexanal also increases the level of trust during collaboration with a social robot. In a preregistered double-blind, placebo-controlled study, we tested if trial-by-trial and general trust during perceptual decision making in collaboration with a social robot is affected by hexanal across two samples (*n* = 46 and *n* = 44). It was hypothesized that unmasked hexanal and hexanal masked by eugenol, a molecule with a smell resembling clove, would increase the level of trust in human–robot interaction, compared to eugenol alone or a control condition consisting of only the neutral smelling solvent propylene glycol. Contrasting previous findings in human interaction, no significant effect of unmasked or eugenol-masked hexanal on trust in robots was observed. These findings indicate that the conscious or nonconscious impact of smell on trust might not generalise to interactions with social robots. One explanation could be category- and context-dependency of smell leading to a mismatch between the natural smell of hexanal, a smell also occurring in human sweat, and the mechanical physical or mental representation of the robot.

## Introduction

Trust is central to effective interactions and collaborations with robots [1, 2]. The ability to establish and sustain trust in a robot determines the functionality and success of the interaction between humans and robots [1]. Too much trust may lead to over-reliance in the capabilities of the robot, whereas too little trust leads to under-utilisation [3]. Given the important role of trust in human–robot interaction, an understanding of how distinct social cues shape trust behaviour is vital. Trust between agents is modulated by a myriad of social cues, including smell [4, 5]. Hexanal, a smell resembling the smell of freshly cut grass, can increase interpersonal trust [6]. Here, we ask if hexanal can increase trust behaviour during human–robot collaboration.

Trust during human–robot interaction can be defined as “the attitude that an agent will help achieve an individual’s goals in a situation characterized by uncertainty and vulnerability” [2, p. 54]. Sanders and colleagues [7] argue that four factors are important determinants of trust in human–robot interaction: robot performance, robot reliance/human compliance, individual differences, and collaboration. Previous research focused on influencing trust via alterations of the robot, e.g., introducing more human-like features or changing other aspects of its appearance [8]. However, trust is not only directly mediated by the features and behaviour of a robot by itself, but also by the human perception of these features and behaviours while interacting with a robot [1, 9]. One effective way to manipulate trust during human–robot interaction is to focus on a feature of the robot that can be easily changed with the current technological advances *and* is an established social communication channel in humans, namely smell.

Social communication is established by the intentional or unintentional release of sensory signals or cues by a sender, in different communicative channels, for example speech, gestures, bodily posture, facial expression, touch, and indeed, chemosensory (i.e., smell) signals. A perceiver processes these signals or cues explicitly or implicitly and acts by changing their behaviour (including emotions) or attitude [10–12]. People process perceived social cues from the environment, and these cues seem to be transmitted in every sensory modality [e.g., 13]. Social communication via the olfactory channel, or *social chemosignaling*, has gained interest in recent years. The impact of our sense of smell is often being underestimated, although over the last years research has uncovered that humans have a remarkably acute and perceptive sense of smell [14–16]. Humans are able to convey messages of gender, disease, personality, and emotions via their body odour, that other individuals can (subconsciously) perceive and act upon [e.g., 12, 17–19]. Chemical signals are part of the broad array of channels that humans use to communicate socially, and our sense of smell may be one of the factors that can shift the balance of trust in cases where the collaboration is new or ambiguous.

Previous research has highlighted the potential role of one particular group of molecules in human social communication: aldehydes [20, 21]. Aldehydes appear frequently in a compendium of 1840 volatile organic compounds emanating from the human body [22] and are named as among the 25 compounds most frequently isolated from the mixture of molecules found on the human skin [23]. Aldehydes are responsible for the attractive smell of many fruits and flowers like lavender [24–26], and are present in many highly appreciated foods [27]. One aldehyde in particular, hexanal, was previously found to increase interpersonal trust between humans [6]. In that study, participants played a trust game with a virtual opponent, while exposed to different smell conditions. Results showed that when participants were exposed to hexanal (resembling freshly cut grass), participants transferred more money, indicative of increased trust, as compared to a control condition where participants were exposed to eugenol (resembling clove). This effect of hexanal was even found when hexanal was masked by eugenol, so that the experimental and control condition were indistinguishable by smell and indicative of both a conscious and nonconscious effect of hexanal on trust. In a follow-up experiment, participants who were exposed to hexanal, rated ambiguous faces as being more trustworthy [6]. This set of studies suggests that hexanal can increase interpersonal trust in human settings. To what extent the trust enhancing effect of smell generalises across situations and agents remains unknown.

In this preregistered double-blind, placebo-controlled study, we ask the question if by manipulating a global feature of the robot via the established social communication channel of smell, trust behaviour can be increased during human–robot collaboration. In a collaborative perceptual decision-making paradigm, the number of answers changed by the human to the answer of the robot’s, was taken as reflecting trust on a trial-by-trial basis. General levels of trust were measured by an established self-reported rating of trust, the Reliance Intention Scale [31]. This study presents a conceptual replication of previous work [6], and is directly replicated by collecting two data samples in parallel. Based on Van Nieuwenberg et al. [6] it is hypothesized that hexanal and hexanal masked by eugenol will increase the trust relationship between humans and robots compared to the no smell condition, while other smell conditions (eugenol alone) will not increase trust compared to the no smell condition. Specifically, we hypothesized that both unmasked hexanal (H1) and hexanal masked by eugenol (H2) will increase the trust-relationship between humans and robots, while the smell of eugenol will not affect the trust-relationship between humans and robots (H3).

## Method

### Data Statement

The preregistration, data, materials, and code are publicly available on the Open Science Framework, https://osf.io/ys8na/. We report all measures in the study, all manipulations, any data exclusions, and the sample size determination rule.

### Participants

An a-priori power-analysis was done to determine the required sample size. This was based on previous work [6], who found, using a between-subjects design with 82 participants, an effect of hexanal masked by eugenol of *d* = 0.79 compared to eugenol alone. This effect is equivalent to an eta-squared of 0.13, i.e., a medium to large effect. Using a within-subjects design, we aimed to include 45 participants, enough to find much smaller effects, as can be expected in human–robot interaction. We then doubled the amount of participants (amounting to *n* = 90). This made sure that a direct replication of the results was possible, while simultaneously allow the two groups of students to collect their own sample as part of their BSc-thesis. Participants were all recruited at the same time, through advertising the study in the local university participant pool and via social media. Participants with odd subject numbers were allocated to sample 1, and participants with even subject numbers were allocated to sample 2. Subject numbers were determined following entry order. In total, 90 participants participated in the study allocated two samples collected in parallel. Forty-six participants, nine men and thirty-seven non-pregnant women, aged 18 to 30 (Mage = 21.8 years, SD = 1.8, age range = 19–28 years) were recruited as part of sample 1.. Forty-four participants were included in sample 2, of which 13 male, 31 female. The participants were aged 19 to 29 (M_age_ = 21.7 years, SD_age_ = 2.0). Written consent was obtained from all participants and the study was in accordance with the guidelines of Utrecht University’s Faculty Ethics Review Board. The protocol was approved by Utrecht University’s Faculty Ethics Review Board, under number 21–1272. Participants not able to participate due to COVID-19 symptoms or because they did not meet the criteria of the screening (including having normal sense of smell) were not included. Following data preparation one participant was excluded from analyses regarding the OpenSesame component of the experiment (number of changed answers and smell ratings) due to an error in the data file. At the start of the experimenting phase, nine participants were tested with an older version of the OpenSesame experiment. This resulted in missing data concerning smell intensity, pleasantness, and familiarity due to an error while saving, but their data was used to test the main hypotheses, resulting in a final sample size of *n* = 46 for sample 1 and *n* = 44 for sample 2.

### Design

A repeated-measures design was used with smell condition (Hexanal, Hexanal masked by Eugenol, Eugenol, no smell) as within-subjects factor and trial-by-trial trust decisions, measured by number of answers changed to the Robot’s answer during a Collaborative perceptual decision-making task and general levels of trust, as measured by the Reliance Intention Scale, as dependent variables. Two samples were collected in parallel and a Latin-square design was used for counterbalancing.

## Materials

### Odours

The odour stimuli were based on the research of Van Nieuwenburg et al. [6]. Unmasked hexanal consisted of 0.01% hexanal (1 μl; CAS 66-25-1; 98% purity, Sigma-Aldrich) diluted in propylene glycol 9.999 ml. Hexanal masked by eugenol (10 ml) consisted of 10% eugenol (1 ml) and 0.01% hexanal (1 μl), diluted in 89.99% propylene glycol (8.999 ml). The masking odour was also presented as a control condition: 10% eugenol (1 ml; CAS 97–53-0.; 99% purity, Sigma-Aldrich) and 90% propylene glycol (9 ml). In addition, a nearly odourless condition consisting of only the dilutant propylene glycol (10 ml) was also included. The odours, each consisting of 10 ml solution, were presented in a 250 ml transparent Duran glass bottle with DIN thread and cap, and labelled with a random, 3-digit code on the exterior. Both the participants and the experimenters were not aware of the coding system. This double-blind design minimized the chance of an experimenter bias. The odours were stored in a refrigerator to minimize evaporation and taken out of the refrigerator at least half an hour prior to the experiment to ensure room temperature during the experiment. Stimuli were replaced after every five participants. There was a minimum of five hours in between the use of the same odour bottles to ensure sufficient headspace. Participants rated the odours for pleasantness (1: very unpleasant, to 7: very pleasant), intensity (1: very weak, to 7: very strong) and familiarity (1: very unfamiliar, 7: very familiar).

### Robot

A commercially available robot named Vector (Anki, San Fransicsco, CA USA / Digital Dream Labs, Pittsburgh, PA USA) was used in this study. Vector is a palm-sized entertainment robot (3.93 × 2.36 × 2.73 inches) with a small head with LED display, fork, and tracks, that features emotional expressions in movement and voice. During this experiment Vector was limited in its movement, but was still able to react to petting, being put on its side, and made noises. Vector was placed on the charging station without the rubber bands normally wrapped around its tracks, to prevent Vector from driving around the lab. When odour bottles were exchanged Vector was shortly petted or removed from the charging station to ensure its alertness during the condition and prevent Vector from going into snooze mode.

### Collaborative Perceptual Decision-Making Task

**Fig. 1:**
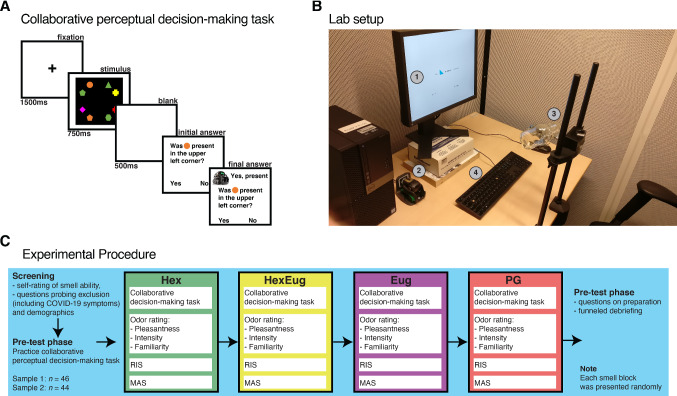
Panel with visual explanation of the experimental procedure, with in **1A:** the collaborative perceptual decision-making task. To measure trial-by-trial trust, an collaborative perceptual decision-making task was used adapted from [28, 29]. Participants saw a complex configuration of visual symbols and indicated whether they had seen one specific symbol. After providing their initial answer they had the opportunity to switch their answer to that of the robot. Note that the task was executed in Dutch. 1B. Overview of the lab setup. In (1) the computer on which the participants saw the task. Vector is visible at (2). The smell stimulus was clamped to the chin-rest that the participant was instructed to place their head in (3). Participants responded using the keyboard marked with (4). In 1C a visual overview of the entire procedure

A customized version of a collaborative perceptual decision-making task was used to assess the extent to which a participant trusted the robot. This visual task was based on the research of Di Lollo, Enns and Rensink [28] and of Kahan and Mathis [29]. Participants were shown a screen for 750 ms with eight shapes in different colours and asked whether a specific shape in a specific colour was present in a particular location on the screen. In total, there were eight different shapes, eight different colours and 4 different locations (quadrants). Each shape and colour could be present multiple times within one trial. After giving their answer, participants were presented with the answer of Vector. The answers given by Vector were correct two-thirds of the time, but participants were not aware of this fact. After receiving this answer, participants could give their definitive answer. See Fig. 1A for a visual overview of this task. The level of trust is measured by the number of times the participant altered their answer to the answer given by the Vector robot. Participants did not receive any feedback on their own performance or that of Vector. This was done to discourage a learning effect throughout the conditions, to enable the use of multiple trials in a game concerning social trust, and to exclude feedback as a possible confounder. Each condition contained fifteen trials. The experiment was programmed in OpenSesame [30].

### Questionnaires

After each condition, the Reliance Intention Scale (RIS) by Lyons and Guznov [31] was presented in Qualtrics (Qualtrics, Provo, UT). Lyons and Guznov specifically adjusted the RIS to assess the level of trust in robots by measuring the anticipated, trust-related attitudes and behaviours towards the robot. The RIS was chosen to assess trust in the robot because of the results in a study by Chita-Tegmark et al. [32]. In this study [32], four questionnaires that measure trust in human–robot interaction were compared. The RIS scored best, as no items were labelled “Not applicable to robots in general". Chita-Tegmark and colleagues replaced the terms “the/this system” in the questionnaire with the term “the robot”, as was done in this study. The RIS consists of 10 items (i.e., "I really wish I had a good way to monitor the decisions of the robot"). The answers were given on a 7-point Likert scale: “Strongly disagree", "Disagree", "Somewhat disagree", "Neither disagree nor agree", "Somewhat agree", and "Strongly agree". In the original study [31], the Cronbach's alpha was 0.83 for the RIS. We found a Cronbach’s alpha of 0.92, indicating high internal consistency and thus reliability of the questionnaire. The RIS was translated to Dutch.

After filling in the RIS, participants were presented with the Mood Arousal Scale [MAS; 33]. The MAS measures (potentially odour-induced) feelings by presenting participants with six items (i.e., “sad—happy”) in directionally counterbalanced orders on 10-point scales. This was done to control for possible odour-induced changes in implicit positive or negative effect. The questionnaire indicates whether the effect of the odours on trust is driven by changes in general implicit affective state. In the current application, a Cronbach’s alpha of 0.72 for the Mood Scale and a Cronbach’s alpha of 0.94 for the Arousal Scale was found, indicating high internal consistency and thus reliability of the questionnaire. The MAS was translated to Dutch.

### Procedure

Potential participants first completed a screening questionnaire containing demographic information (e.g., age, gender, pregnancy), a self-rating of smell ability, questions probing exclusion criteria based on [6] and [34], and information concerning COVID-19 symptoms. Participants were provided with additional information regarding the experiment and, when eligible, invited to come to the laboratory. One day before the actual experiment, participants were contacted again via e-mail to remind them of the preparations: refrain from drinking alcohol and smoking 24 h before the experiment; avoid using lotions, creams, cosmetics, and deodorant on the day of the experiment; no consumption of any caffeinated drinks 3 h prior to participation; and no exercise one hour before participation.

At the start of the experiment, the participant was escorted to the lab, where the experiment was explained. After receiving this information, the participant provided written consent. At the start of the experiment, Vector was introduced as a modern and advanced robot and the participant was instructed to pet Vector and put Vector on its side to elicit an emotional reaction by Vector. The participant was then told that Vector was programmed to cooperate with them on the task. The interaction with Vector lasted a maximum of two minutes per participant. Vector was then placed on its docking station, for which the participant was told that it was connected to the computer to Vector to collaborate. Vector was positioned, next to the monitor within eyesight of the participant during the experiment. As part of the cover story, it was explained that Vector was connected to the computer and participated in the task by interacting with the computer directly. In reality, there was no interaction between the computer and robot, and the task was set up in such a way that Vector’s answers were pre-determined by the experimental task.

Testing was done in a test cubicle of 4 m^2^ (air circulation: 5 cycles/h), similar to [6]. The bottle containing the odour stimulus was placed 3 cm below the nose of the participant with an extension clamp that was attached to a head-and-chin rest. Participants were aware that the bottle could contain a smell, and were asked to evaluate the smell at the end of every condition. The participant executed the task on a monitor (60 Hz) placed 50 cm from the participants’ eyes, ensured by the chinrest. See Fig. 1B for a picture of the experimental set-up. The visual detection task was explained using written instructions and the participant practiced eight trials in the absence of an odour bottle. The participant was given the opportunity to ask questions. Once it was confirmed that the participant understood the task, the main task started.

Each condition consisted of 15 trials of the collaborative perceptual decision-making task with three follow-up questions regarding the pleasantness, intensity, and familiarity of the smell. After each condition, the participant was asked to leave the test room so that the experimenter could install the new odour. This was repeated for all four conditions. The RIS and MAS were answered on a computer in the waiting room while the odour was replaced by the experimenter. At the end of all trials the participants received some additional questions regarding their preparation on the day of the experiment (e.g., use of lotions, caffeine). In total, the experiment lasted 30 to 45 min. Afterwards the participants were debriefed, given the opportunity to ask questions and were thanked for their participation. Ten gift cards worth 25 euros were raffled to participants who indicated that they wanted to be eligible for this compensation.

Due to the COVID-19 pandemic that was ongoing when this experiment was carried out, participants were asked a brief health check before entering the lab, and the experimenter and participant kept 1,5 m distance at all times to prevent spreading of the virus. The experimenter wore gloves when handling stimulus materials. After each condition, the participant was asked to leave the experiment cubicle to enable the experimenter to replace the odour bottle. Meanwhile, the participants filled in the questionnaire about trust and mood arousal on the desktop in the waiting room. After each participant, all surfaces touched during the experiment were cleaned using cleaning wipes by WypAll, from Kimberley-Clark Professional. This included the armrests and seat height adjusters of the chairs, the keyboards and mouses of the computers, the robot, the chin rest, the doorknobs, the pens, the tables, and the exterior of the odour bottles. This was done at least ten minutes before arrival of the next participant.

### Data-Analysis

Besides a repeated-measures ANOVA with condition (4) as within-factor preregistered as part of undergraduate training, we preregistered a generalised mixed-effects logistic regression model to test the impact of smell on trust decisions, i.e., number of times participants changed their answers to the robot’s answer (binomial dependent variable). We used contrast coding for smell condition (condition_smell.f) to test the three hypotheses. To test a general effect of hexanal on trust (hypotheses 1 and 2), the first contrast compared Hexanal and Hexanal masked by Eugenol with Eugenol and the no smell condition [Hexanal: + 1/2, Hexanal masked by Eugenol: + 1/2, Eugenol: –1/2, no smell: –1/2]. To test if eugenol did not affect the trust-relationship between humans and robots, the second contrast compared Hexanal and Hexanal masked by Eugenol with Eugenol [Hexanal: + 1/2, Hexanal masked by Eugenol: + 1/2, Eugenol: –1, no smell: 0]. The third contrast compared Hexanal with Hexanal masked by Eugenol [Hexanal: + 1/2, Hexanal masked by Eugenol: -1/2, Eugenol: 0, no smell: 0], to test the impact of Hexanal masking. Following [51], we started with a maximum random effects structure and systematically reduced the complexity until convergence. The following generalised mixed-effects logistic regression model, with smell condition as a fixed effect (contrast coded), by-subject and by-round random intercepts successfully converged for sample 1:

$$ changed\_to\_vector \, \sim \, condition\_smell.f \, + \, \left( {1|subject\_nr} \right) \, + \, \left( {1 | \, round} \right) $$

For sample 2, the following model, with smell condition as a fixed effect, by-subject, by-trial, and by-round random intercepts successfully converged:

$$ changed\_to\_vector \, \sim \, condition\_smell.f \, + \, \left( {1|subject\_nr} \right) \, + \, \left( {1 \, | \, trial} \right) \, + \, \left( {1 | \, round} \right) $$

To test an effect of smell on general levels of trust, a linear model was fitted with smell condition (contrast coded) as fixed effect.

We also report Bayesian posterior distributions as an additional source of information about the evidence for the alternative hypothesis versus the null-hypothesis given the data.

## Results

To confirm that conditions did not differ in the amount of induced arousal or induced changes in mood, the scores on the MAS were compared using two repeated measures ANOVAs. There was no effect of the different odour conditions on mood, sample 1 *F*(3, 129) = 0.55, *p* = 0.645, η_p_2 = 0.01; sample 2 *F* (3, 129) = 2.07, *p* = 0.107, η_p_2 = 0.05. There was no effect of odour condition on arousal, sample 1 *F* (3, 129) = 0.42, *p* = 0.739, η_p_2 = 0.01; sample 2 *F* (3, 129) = 0.72, *p* = 0.545, η_p_2 = 0.02.

**Fig. 2:**
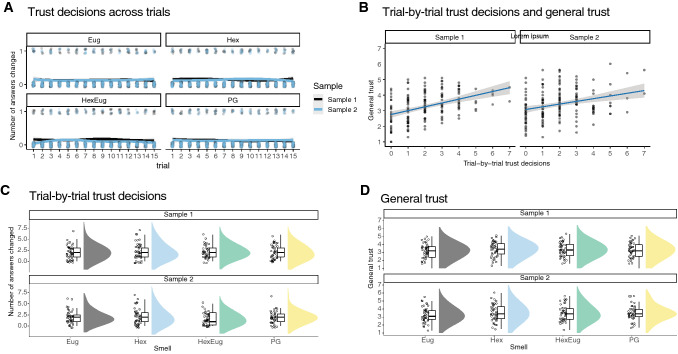
No effect of smell on trial-by-trial trust decisions and general trust. Trial-by-trial trust decisions, as measured by number of answers changed to the Robot’s answer, remained constant throughout the trials for each smell block (**A**). Trial-by-trial trust decisions and general trust, as measured by the reliance intention scale (RIS) were positively correlated (**B**). No effect of Hexanal nor Hexanal masked by Eugenol was found on trial-by-trial trust decisions (**C**) and general trust, as measured by the reliance intention scale (RIS) (**D**). No differences were observed between the smell conditions for both measures of trust. Individual answers per participants and trial are visualised in A and coded as 1: changed, 0: not changed, with a nonparametric smoothed curve added to indicate overall trends. Average general trust and total number of changed answers indicative of trial-by-trial trust per participant are visualised in B, while rain clouds plots with errors bars reflecting 95% confidence intervals are shown in C and D [50]. Eug: Eugenol, Hex: Hexanal. HexEug: with Hexanal masked by Eugenol, PG: propylene glycol

During the collaborative perceptual decision-making task, participants changed their answers on average on 2.1 (sample 1) and 1.9 (sample 2) out of 15 trials. Decisions to trust the robot were consistent through the rounds of the collaborative perceptual decision-making task (Fig. 2A). Average level of trust of the robot was 3.3 (sample 1) and 3.4 (sample 2) on a 7-point scale. Trial-by-trial trust decisions and general trust were positively associated (repeated measures correlation across participants and conditions [52], sample 1: *r*(124) = 0.14, [−0.04, 0.31], *p* = 0,14; sample 2: *r*(139) = 0.26, [0.10, 0.41], *p* = 0.034,), confirming the validity of the operationalization of trust (Fig. 2B).

Hexanal and Hexanal masked by Eugenol did not increase trust decisions on a trial-by-trial basis compared with Eugenol and the no smell condition (sample 1: *β* = 0.05, *p* = 0.80, 95%*CI* = [−0.37, 0.47]; sample 2: *β* = 0.01, *p* = 0.95, 95%*CI* = [−0.42, 0.45]), or Eugenol alone (sample 1: *β* = 0.07, *p* = 0.69, 95%*CI* = [−0.28, 0.43]; sample 2: *β* = −0.09, *p* = 0.60, 95%*CI* = [−0.44, 0.26]) (Fig. 2C). Directly contrasting Hexanal with Hexanal masked by Eugenol showed that for sample 2 (*β* = 0.20, *p* = 0.03, 95%*CI* = [0.02, 0.37]), but not for sample 1 (*β* = −0.03, *p* = 0.71, 95%*CI* = [−0.20, 0.14]), trial-by-trial trust decisions were higher for Hexanal compared with the Hexanal masked by Eugenol. Bayesian analysis revealed similar results with moderate support for the null hypothesis (See online supplementary files on https://osf.io/ys8na/).

Hexanal did not impact general levels of trust ascribed to the robot, as measured using the Reliance Intention Scale (Fig. 2D). Hexanal and Hexanal masked by Eugenol compared with Eugenol and the no smell condition (sample 1: *β* = 0,43, *p* = 0.07, 95%*CI* = [−0.03, 0.89]; sample 2: *β* = 0.27, *p* = 0.19, 95%*CI* = [−0.14, 0.67]), or Eugenol alone, (sample 1: *β* = −0,25, *p* = 0.18, 95%*CI* = [−0.63, 0.12]; sample 2: *β* = −0.14, *p* = 0.40, 95%*CI* = [−0.47, 0.19]). Directly contrasting Hexanal with Hexanal masked by Eugenol showed that general trust ratings were higher for Hexanal compared with the Hexanal masked by Eugenol for sample 1 (*β* = −0.21 *p* = 0.03, 95%*CI* = [−0.39, −0.02]), but not for sample 2 (*β* = −0.09, *p* = 0.29, 95%*CI* = [−0.25, 0.08]). Bayesian analysis revealed similar results with moderate support for the null hypothesis (See online supplementary materials on https://osf.io/ys8na/).

Together, these results contrast with our predictions: Neither Hexanal nor Hexanal masked by Eugenol increases trial-by-trial trust decisions during collaborative perceptual decision-making or general levels of trust.

Finally, to check whether the conditions differed in pleasantness, familiarity and intensity, the odour ratings for each condition were compared by means of three repeated measures ANOVAs, with condition as factor and the ratings as dependent measure. Due to an error in the programming of the experiment, these values were not recorded for the first few participants. This resulted in different sample sizes for this analysis: *n* = 43 for sample 1, and *n* = 40 for sample 2.

There was a difference in the ratings for pleasantness in sample 2, Sample 1: *F*(3, 123) = 1.0, *p* = 0.397, η_p_^2^ = 0.02; sample 2: *F*(3, 117) = 11.2, *p* < 0.001, η_p_^2^ = 0.22. In sample 2, hexanal was rated as more pleasant than hexanal masked by eugenol condition (mean difference = 0.90, SE = 0.29), *p* = 0.019, and hexanal was rated as more pleasant than eugenol (mean difference = 1.28, SE = 0.31), *p* = 0.001. Propylene glycol was also rated as more pleasant than eugenol (mean difference = 1.15, SE = 0.23), *p* < 0.001. Critically, there was no difference between the condition pairs pertinent for the hypotheses tested in the study, i.e., hexanal and propylene glycol, and hexanal masked by eugenol and eugenol alone.

There was a difference in the ratings for intensity; sample 1: *F*(3, 117) = 32.9, *p* < 0.001, η_p_^2^ = 0.46; sample 2: (3, 117) = 38.9, *p* < 0.001, η_p_^2^ = 0.50. Bonferroni corrected pairwise comparisons suggested that the eugenol conditions were significantly more intense than the other two conditions, *p’*s < 0.001. Critically, the propylene glycol condition did not differ in intensity from the hexanal condition (mean difference = 0.45, SE = 0.32), *p* > 0.05, nor did the eugenol and hexanal masked by eugenol condition differ from each other (mean difference = 0.28, SE = 0.19), *p* > 0.05. While there was a difference in the ratings of familiarity in sample 1, *F*(3, 123) = 2.98, *p* = 0.034, η_p_^2^ = 0.07, there was no difference in familiarity ratings for sample 2, *F*(1, 117) = 1.51, *p* = 0.217, η_p_^2^ = 0.04. No pairwise comparisons were significant after Bonferroni correction for multiple testing.

## Discussion

The aim of this study was to investigate the impact of smell on trust during human–robot collaboration. As a conceptual replication of previous work during human interaction [6], we tested the influence of hexanal on trust ascribed to a social robot during collaborative perceptual decision-making in two samples. In contrast to our predictions, we did not observe a hexanal-mediated increase in both trial-by-trial trust decisions and general trust during human–robot collaboration. Neither masked nor unmasked hexanal influenced trust compared to the control conditions. While hexanal increased interpersonal trust between humans [6], we did not observe an increase in trust during human–robot collaboration. One explanation of the contrasting results is that hexanal might work in a context-dependent manner. In general, many effects of smells on human behaviour have been found to be dependent and modulated by context. For instance, in one now famous study, participants rated the pleasantness of the same odour mixture as pleasant or unpleasant, depending on whether it was labelled either ‘parmesan cheese’ or ‘vomit’ (i.e., a linguistic context; [35]). In a similar study, the provided label context of a diverse array of smells was found to influence physiological reactions to those smells in addition to rated pleasantness, including skin conductance and sniff volume [36]. While in a social context with a human agent, hexanal would increase interpersonal trust, this effect might disappear when the interaction takes place with an artificial agent. As hexanal occurs in human sweat [20] and serves as a social cue, it can be paired to trustworthy others via associative learning. Removing one part of this context, i.e., the human social interaction, would then mitigate the effect.

Perceiving and interacting with social robots is determined by an interplay between visual features of the robot, such as the shape and size, and knowledge factors within the human observer, such as expectations and beliefs [9]. A similar breakdown has been made for trust during human–robot interaction, with factors related to the robot, human user, and task and environment, being critical in determining trust [37, 38]. A small-scale meta-analysis [37] suggested that out of these three factors, the features of the robot, in particular its performance, are influential in determining trust during collaborations with a robot. The perception of the performance of the robot can interact with expectations held by the human user. If misaligned, for instance if the expectations of the user are high but the performance of the robot is poor, an expectation gap might appear [39]. Lower trust can be the result of not only low reliability of the robot [40], but also unmet expectations [41, 42]. Interpersonal trust is a multifaceted phenomenon dependent on a myriad of factors, and a focus beyond performance-, behaviour-, or appearance-related factors is warranted to explore new ways to facilitate optimal trust during human–robot interaction.

Trust can be operationalised and measured in various ways. Here, we assess trust on two levels by measuring trial-by-trial trust decisions during a collaborative perceptual decision-making task and general trust as measured by an established questionnaire. These measures have successfully been used in previous human–robot studies [e.g., 28, 29, 32], and were found to correlate moderately in the present study. However, we did not observe an effect of hexanal on these measures. The possibility exists that hexanal may have much more subtle effects on trust beyond the range of these tasks. One way to improve the sensitivity of the task in future studies could be to increase the number of trials on which there is an opportunity to change the answer to that of collaborating robot, although this could have attrition effects on the participant’s attention. Another possibility to further test the hypothesis that hexanal can influence human–robot trust, is to operationalize human–robot trust in a different way. Van Nieuwenburg and colleagues [6] used an ambiguous face paradigm and a single trial trust game. Both tasks might be more sensitive to establishing changes in trust than the current measures.

For humans, the sense of smell drives approach and avoidance behaviour, serves as a guide to what we eat, and has a social function [43, 44]. Human body odours contain information on who we are, what emotions we are feeling, and whether we are healthy or not. This information has communicative value for other people [e.g., 12]. Both the general public and the scientific community underestimates the power of smell. The human sense of smell is much better than most people think and olfaction is often neglected as a modality for scientific research, with its traditional strong focus on vision and audition [e.g., 14, 15]. This ignores the role olfaction plays in human social interaction. With increasing insights in the role of smell, the potential that olfaction may offer to improve communication in social contexts can be explored, for example to improve trust between humans and robots. The important roles olfaction as a primary sense play in our daily lives justifies more attention.

Before dismissing the effect of hexanal on trust, or on human–robot trust, completely, several alternatives should be considered. First, a closer replication of the previous study [6], using a trust game with a human and robot condition, or using an ambiguous face task, should be considered. Second, other odours may be considered to test their effect on trust of robots. Natural odours, including body odours, are usually highly complex mixtures of tens or hundreds of molecules [19, 45, 46]. A more ecological approach would be to test the effect of hexanal within a mixture of odours, such as the smell of lavender that has previously also been used to improve interpersonal trust [4]. Counter to this, would be the possibility to test the category- and context-dependency by using more artificial odour mixtures, such as *the smell of data,* an odour specifically designed to serve as a warning signal in case of data leaks by olfactory artists Leanne Wijnsma and Froukje Tan [47]. Another potential bias in the current study paradigm was a misattribution of the smell to the robot: participants may have attributed the smell conditions to the experiment, or to the room, instead of to the robot, mitigating a potential effect. Although this explanation is not very likely, since the same procedure was used in the study by Van Nieuwenburg et al. [6], future studies should consider to directly scenting the robot instead of using ‘external’ fragrances. Finally, cross-platform generalisability [48, 49] could play a role. In a recent evaluation of trust questionnaires most often employed in human–robot interaction, issues of generalisability have been reported [32]. Items used in a questionnaire not necessarily generalise across robots or situations. This offers the possibility that a complex interaction between odour, robot, and trust measure might explain the results. Together, these considerations offer great opportunities for future studies to employ context- and category-dependent odour-robot combinations using a multidimensional assessment of trust. For instance, a study could investigate the effects of a human- and machine-like robot paired with natural and synthetic odour.

In conclusion, we did not observe an effect of hexanal on trust in human–robot collaboration, which was operationalized using two different dependent measures. A category- and context-dependency could potentially underlie these results, whereby the associative learning linked to social chemosignaling mismatches with the cognitive representations of the robot. Since smell is a means to communicate a diverse array of signals, social chemosignaling offers a new vista for human–robot interaction and to enable fluent collaboration between robots and humans. The current research is a first endeavour into how the sense of smell is potentially involved in human–robot interaction and highlights the need for more research into the social functions of smell more broadly.

## Data Availability

Data and materials can be found on https://osf.io/ys8na/files/.
